# Supplementary material for: A game theoretic approach reveals that discretizing clinical information can reduce antibiotic misuse
Source: Nat Commun. 2021 Feb 19;12:1148. doi: 10.1038/s41467-021-21088-5 (PMC7895914; doi:10.1038/s41467-021-21088-5)
Supplement: Supplementary file 1 — Supplementary Information [file 41467_2021_21088_MOESM1_ESM.pdf]

Supplementary Information for A game theoretic approach reveals that discretizing clinical information can reduce antibiotic misuse

Supplementary Note 1 Extended calculation of Payoffs and MPE with a Dichotomous Signal System

If the strategy profile is symmetric, then the payoff of each player when they both treat everyone ($d^k = 0$) is:

$$\begin{aligned} v_i^k\left(s^{k-1},0,0\right) &= k\alpha(r_2 - r_1)\int_0^1 p \cdot f(p)dp + v_i^{k-2}\left(s^{k-2}\right) \\ &= k\alpha(r_2 - r_1)(F(T)p_L + [1 - F(T)]p_H) + v_i^{k-2}\left(s^{k-2}\right) \end{aligned}$$

and when both treat only patients with a high signal ($d^k = T$) it is:

$$\begin{aligned} v_i^k\left(s^{k-1},T,T\right) &= \left(k\alpha(r_2 - r_1)\int_T^1 p \cdot f(p)dp + 2F(T)\left(1 - F(T)\right)v_i^{k-1}\left(s^{k-1}\right) + \left(1 - F(T)\right)^2 v_i^{k-2}\left(s^{k-2}\right)\right. \\ &\quad \cdot \frac{1}{1 - \left(F(T)\right)^2} \\ &= \quad k\alpha(r_2 - r_1)(1 - F(T))p_H + 2F(T)\left(1 - F(T)\right)v_i^{k-1}\left(s^{k-1}\right) + \left(1 - F(T)\right)^2 v_i^{k-2}\left(s^{k-2}\right) \\ &\quad \cdot \frac{1}{\left(1 - F(T)\right)\left(1 + F(T)\right)} \\ &= \quad k\alpha(r_2 - r_1)p_H + 2F(T)v_i^{k-1}\left(s^{k-1}\right) + \left(1 - F(T)\right)v_i^{k-2}\left(s^{k-2}\right) \quad \cdot \frac{1}{1 + F(T)} \end{aligned}$$

If the strategy profile is not symmetric, then the payoff of the physician who treats everyone is:

$$\begin{aligned} v_i^k\left(s^{k-1},0,T\right) &= k\alpha(r_2 - r_1)\ {}_0^1\ p \cdot f(p)dp + F(T)v_i^{k-1}\left(s^{k-1}\right) \\ &\qquad +(1 - F(T))v_i^{k-2}\left(s^{k-2}\right) \\ &= k\alpha(r_2 - r_1)(F(T)p_L + [1 - F(T)]p_H) + F(T)v_i^{k-1}\left(s^{k-1}\right) \\ &\qquad +(1 - F(T))v_i^{k-2}\left(s^{k-2}\right) \\ &= F(T)\left[k\alpha(r_2 - r_1)p_L + v_i^{k-1}\left(s^{k-1}\right)\right] \\ &\qquad +[1 - F(T)]\left[k\alpha(r_2 - r_1)p_H + v_i^{k-2}\left(s^{k-2}\right)\right] \end{aligned}$$

and the payoff of the physician who treats only patients with a high signal is:

$$
\begin{aligned}
v_i^k\left(s^{k-1},T,0\right) &= k\alpha(r_2-r_1)\ {}_{T}^{1}\, p\cdot f(p)dp + F(T)v_i^{k-1}\left(s^{k-1}\right) \\
&\qquad +(1-F(T))v_i^{k-2}\left(s^{k-2}\right) \\
&= k\alpha(r_2-r_1)[1-F(T)]p_H + F(T)v_i^{k-1}\left(s^{k-1}\right) \\
&\qquad +(1-F(T))v_i^{k-2}\left(s^{k-2}\right) \\
&= F(T)\left[v_i^{k-1}\left(s^{k-1}\right)\right] \\
&\qquad +[1-F(T)]\left[k\alpha(r_2-r_1)p_H + v_i^{k-2}\left(s^{k-2}\right)\right]
\end{aligned}
$$

When we want to check whether a given symmetric strategy profile is an MPE, the One-Stage Deviation Principle still applies, but there are only two types of possible deviations - either from $d^k = T$ to $d^k = 0$ or vice versa. Thus, $d^k = 0$ is a symmetric stage equilibrium iff:

$$
v_i^k\left(s^{k-1},0,0\right) \geq v_i^k\left(s^{k-1},T,0\right)
$$

Using (R.1) and (R.4) we get

$$
\begin{aligned}
&k\alpha(r_2-r_1)(F(T)p_L + [1-F(T)]p_H) + v_i^{k-2}\left(s^{k-2}\right) \geq \\
&F(T)\left[v_i^{k-1}\left(s^{k-1}\right)\right] + [1-F(T)]\left[k\alpha(r_2-r_1)p_H + v_i^{k-2}\left(s^{k-2}\right)\right]
\end{aligned}
$$

which means

$$
k\alpha(r_2-r_1)p_L \geq v_i^{k-1}\left(s^{k-1}\right) - v_i^{k-2}\left(s^{k-2}\right)
$$

and $d^k = T$ is a symmetric stage equilibrium iff:

$$
v_i^k\left(s^{k-1},T,T\right) \geq v_i^k\left(s^{k-1},0,T\right)
$$

Using (R.2) and (R.3) we get

$$
\begin{aligned}
&\left(k\alpha(r_2-r_1)p_H + 2F(T)v_i^{k-1}\left(s^{k-1}\right) + \left(1-F(T)\right)v_i^{k-2}\left(s^{k-2}\right)\right)\cdot\frac{1}{1+F(T)} \geq \\
&F(T)\left[k\alpha(r_2-r_1)p_L + v_i^{k-1}\left(s^{k-1}\right)\right] + [1-F(T)]\left[k\alpha(r_2-r_1)p_H + v_i^{k-2}\left(s^{k-2}\right)\right]
\end{aligned}
$$

which means

$$
k\alpha(r_2-r_1)(F(T)[1+F(T)]p_L - [F(T)]^2 p_H) \leq F(T)[1-F(T)]\left[v_i^{k-1}\left(s^{k-1}\right) - v_i^{k-2}\left(s^{k-2}\right)\right]
$$

or

$$\frac{k\alpha(r_2 - r_1)([1 + F(T)]p_L - F(T)p_H)}{1 - F(T)} \leq v_i^{k-1}(s^{k-1}) - v_i^{k-2}(s^{k-2})$$

Supplementary Note 2 Claim R.1

Claim R.1: Under the fixed symmetric policy $s = (T, T)^M$ (*i.e.* $d^k = T, \quad k = 1, \dots, M$)

$$v_i^k(s^k) - v_i^{k-1}(s^{k-1}) > 0, \qquad k = 1, \dots, M$$

That is, the fixed symmetric policy of "treating only patients with a high signal" yields a total expected payoff (from E-state k onwards) that is strictly increasing in k.

Proof. By induction.

By definition, if $k \leq 0$ then $v_i^k = 0$. If $k \geq 1$ then using (R.2):

$$\begin{aligned} & v_i^k(s^k) - v_i^{k-1}(s^{k-1}) \\ & = k\alpha(r_2 - r_1)p_H + 2F(T)v_i^{k-1}(s^{k-1}) \\ & + (1 - F(T))v_i^{k-2}(s^{k-2}) \quad \cdot \frac{1}{1 + F(T)} \\ & - (k-1)\alpha(r_2 - r_1)p_H + 2F(T)v_i^{k-2}(s^{k-2}) \\ & + (1 - F(T))v_i^{k-3}(s^{k-3}) \quad \cdot \frac{1}{1 + F(T)} \\ & = \frac{1}{1 + F(T)} \\ & \cdot \alpha(r_2 - r_1)p_H + 2F(T) \quad v_i^{k-1}(s^{k-1}) - v_i^{k-2}(s^{k-2}) \\ & + (1 - F(T)) \quad v_i^{k-2}(s^{k-2}) - v_i^{k-3}(s^{k-3}) \end{aligned} \tag{S-2.1}$$

(S-2.1) holds for $k = 1,2,3$:

$$v_i^1(T,T) - v_i^0 = v_i^1(T,T) = \frac{\alpha(r_2 - r_1)p_H}{1 + F(T)} > 0$$

$$v_i^2((T,T)^2) - v_i^1(T,T) = \frac{\alpha(r_2 - r_1)p_H + 2F(T)\left(v_i^1(T,T) - v_i^0\right)}{1 + F(T)} = \frac{\alpha(r_2 - r_1)p_H + 2F(T) \quad \frac{\alpha(r_2 - r_1)p_H}{1 + F(T)}}{1 + F(T)} > 0$$

$$v_i^3\left((T,T)^3\right) - v_i^2\left((T,T)^2\right)$$
$$= \frac{1}{1+F(T)}$$
$$\cdot \left[\alpha(r_2 - r_1)p_H + 2F(T)\left(v_i^2\left((T,T)^2\right) - v_i^1(T,T)\right) + \left(1 - F(T)\right)\left(v_i^1(T,T) - v_i^0\right)\right] > 0$$

Assume that *(S-2.1)* holds for some k; then it holds for $k+1$ as well:

$$v_i^{k+1}\left((T,T)^{k+1}\right) - v_i^k\left((T,T)^k\right)$$
$$= \frac{1}{1+F(T)}$$
$$\cdot \quad \alpha(r_2 - r_1)p_H + 2F(T) \quad v_i^k\left((T,T)^k\right) - v_i^{k-1}\left((T,T)^{k-1}\right)$$
$$+ \left(1 - F(T)\right) \quad v_i^{k-1}\left((T,T)^{k-1}\right) - v_i^{k-2}\left((T,T)^{k-2}\right) \quad > 0$$

Supplementary Note 3 Threshold Decision Rules

We present a proof that threshold decision rules are the most efficient, in the sense that for any given non-threshold decision rule we can find a threshold decision rule that yields a higher payoff. First, we need to define a general decision rule.

As defined in section 1.2, a Markovian threshold decision rule is a number $d_i^k \in [0,1)$, which means that physician i will choose A on E-state k (administer antibiotic treatment) if the posterior of his patient is $p_i \geq d_i^k$ and will choose W (withhold treatment) if $p_i < d_i^k$. A Markovian general decision rule is an indicator function $I_i^k: [0,1) \to \{0,1\}$. The value of I_i^k equals 1 for any posterior that physician i wishes to treat. If we define X_i as the set of all posteriors for which physician i chooses A on E-state k, we get:

$$I_i^k(p_i) = \begin{matrix} 1 & \text{if} \quad p_i \in X_i \\ 0 & \text{otherwise} \end{matrix} \qquad \text{(S-3.1)}$$

We assume that I_i^k is measurable. Note that a threshold decision rule is a special case of (S-3.1) with $X_i = \left[d_i^k, 1\right)$.

From the definitions of the payoff function (M.1.5) and (M.1.6) we can see that the decision rule chosen by physician i on E-state k affects his own payoff through two elements: the expected posterior of a patient that is treated by him and the probability that he will indeed treat the patient. The explicit terms of these elements in the case of threshold decision rules are:

$$E_i\left(d_i^k\right) = \int_{d_i^k}^{1} p \cdot f(p)dp \qquad \text{(S-3.2)}$$

$$G\left(d_i^k\right) = 1 - F\left(d_i^k\right) = \int_{d_i^k}^{1} f(p)dp \qquad \text{(S-3.3)}$$

and the explicit terms of these elements in the case of *general* decision rules are (respectively):

$$E_i \quad I_i^k(p_i) \quad = \int_0^1 p \cdot \mathbf{I}_i^k(p) \cdot f(p)dp \qquad \text{(S-3.4)}$$

$$G \quad I_i^k(p_i) \quad = \int_0^1 \mathbf{I}_i^k(p) \cdot f(p)dp \qquad \text{(S-3.5)}$$

Thus, the expected payoff of physician i on E-state k in the case of general decision rules is:

$$\begin{aligned} &v_i^k\left(s^{k-1}, \mathbf{I}_i^k(p), \mathbf{I}_{-i}^k(p)\right) \\ &= \frac{1}{1 - \left[1 - G\left(\mathbf{I}_i^k(p)\right)\right]\left[1 - G\left(\mathbf{I}_{-i}^k(p)\right)\right]} \\ &\cdot \quad kU \cdot E_i\left(\mathbf{I}_i^k(p)\right) + [G\left(\mathbf{I}_i^k(p)\right) \quad 1 - G\left(\mathbf{I}_{-i}^k(p)\right) \\ &+ \quad 1 - G\left(\mathbf{I}_i^k(p)\right) \quad G\left(\mathbf{I}_{-i}^k(p)\right)]v_i^{k-1}(s^{k-1}) \\ &+ G\left(\mathbf{I}_i^k(p)\right)G\left(\mathbf{I}_{-i}^k(p)\right)v_i^{k-2}(s^{k-2}) \end{aligned} \qquad \text{(S-3.6)}$$

Theorem S-3.1:

For any given general non-threshold decision rule I_i^k of physician i there exists a threshold decision rule d_i^k, such that if we define

$$I_i^k(p_i) = \begin{matrix} 1 & \text{if} & d_i^k \leq p_i < 1 \\ 0 & & \text{otherwise} \end{matrix}$$

then $v_i^k\left(s^{k-1}, \mathbf{I}_i^k(p), \mathbf{I}_{-i}^k(p)\right) > v_i^k\left(s^{k-1}, \mathbf{I}_i^k(p), \mathbf{I}_{-i}^k(p)\right)$.

Proof. For any given non-threshold I_i^k we will choose a corresponding d_i^k such that

$$G\left(d_i^k\right) = G\left(\mathbf{I}_i^k(p)\right)$$

and therefore, obviously,

$$G\left(\mathbf{I}_i^k(p)\right) = G\left(\mathbf{I}_i^k(p)\right)$$

By (S-3.6) we get

$$\begin{aligned} &v_i^k\left(s^{k-1}, \mathbf{I}_i^k(p), \mathbf{I}_{-i}^k(p)\right) - v_i^k\left(s^{k-1}, \mathbf{I}_i^k(p), \mathbf{I}_{-i}^k(p)\right) \\ &= \frac{kU \cdot E_i\left(\mathbf{I}_i^k(p)\right)}{1 - \left[1 - G\left(\mathbf{I}_i^k(p)\right)\right]\left[1 - G\left(\mathbf{I}_{-i}^k(p)\right)\right]} - \frac{kU \cdot E_i\left(\mathbf{I}_i^k(p)\right)}{1 - \left[1 - G\left(\mathbf{I}_i^k(p)\right)\right]\left[1 - G\left(\mathbf{I}_{-i}^k(p)\right)\right]} \end{aligned}$$

The denominators are equal by construction, and therefore we need only to compare the nominators. By (S-3.4)

$$kU \cdot E_i\left(\mathbf{I}_i^k(p)\right) - kU \cdot E_i\left(\mathbf{I}_i^k(p)\right) = kU \quad \int_0^1 p \cdot \mathbf{I}_i^k(p) \cdot f(p)dp - \int_0^1 p \cdot \mathbf{I}_i^k(p) \cdot f(p)dp\Bigg]$$

$$\int_0^1 p \cdot I_i^k(p) \cdot f(p)dp - \int_0^1 p \cdot I_i^k(p) \cdot f(p)dp = \frac{\int_0^1 p \cdot I_i^k(p) \cdot f(p)dp}{G\left(d_i^k\right)} - \frac{\int_0^1 p \cdot I_i^k(p) \cdot f(p)dp}{G\left(d_i^k\right)}$$
$$= \frac{\int_0^1 p \cdot I_i^k(p) \cdot f(p)dp}{\int_0^1 I_i^k(p) \cdot f(p)dp} - \frac{\int_0^1 p \cdot I_i^k(p) \cdot f(p)dp}{\int_0^1 I_i^k(p) \cdot f(p)dp}$$

which is the difference in the average posterior per probability of treatment, where both probabilities of treatment are equal $\left(\int_0^1 I_i^k(p) \cdot f(p)dp = \int_0^1 I_i^k(p) \cdot f(p)dp\right)$. Obviously, the highest average posterior per given probability of treatment is achieved by treating the patients with the highest posteriors, and therefore

$$\int_0^1 p \cdot I_i^k(p) \cdot f(p)dp - \int_0^1 p \cdot I_i^k(p) \cdot f(p)dp > 0$$

Supplementary Note 4 Lemma M.2.1

Lemma M.2.1: $\lim_{\epsilon \to 0} v^k(s^{k-1}, 1-\epsilon) = \frac{k\alpha(r_2 - r_1)}{2} + v^{k-1}(s^{k-1})$

Proof.

$$\begin{aligned}
\lim_{\epsilon \to 0} v^k(s^{k-1}, 1-\epsilon) &= \lim_{\epsilon \to 0} \Big(k\alpha(r_2 - r_1) \int_{1-\epsilon}^1 p \cdot f(p)dp \\
&\quad +2F(1-\epsilon)[1 - F(1-\epsilon)]v^{k-1}(s^{k-1}) \\
&\quad +[1 - F(1-\epsilon)]^2 v^{k-2}(s^{k-2})\Big) \cdot \frac{1}{1-\left(F(1-\epsilon)\right)^2} \\
&= \lim_{\epsilon \to 0} \Big(k\alpha(r_2 - r_1)\left[1 - (1-\epsilon)F(1-\epsilon) - \int_{1-\epsilon}^1 F(p)dp\right] \\
&\quad +2F(1-\epsilon)[1 - F(1-\epsilon)]v^{k-1}(s^{k-1}) \\
&\quad +[1 - F(1-\epsilon)]^2 v^{k-2}(s^{k-2})\Big) \cdot \frac{1}{\left(1-F(1-\epsilon)\right)\left(1+F(1-\epsilon)\right)} \\
&= \lim_{\epsilon \to 0} \Big(k\alpha(r_2 - r_1) + 2F(1-\epsilon)v^{k-1}(s^{k-1}) \\
&\quad +[1 - F(1-\epsilon)]v^{k-2}(s^{k-2})\Big) \cdot \frac{1}{1+F(1-\epsilon)} \\
&= \frac{k\alpha(r_2 - r_1)}{2} + v^{k-1}\left(s^{k-1}\right)
\end{aligned}$$

Supplementary Note 5 Theorem M.2.2

Theorem M.2.2: $\lim_{k \to \infty} d^k = 1$

Proof.

Suppose to the contrary that d^k does not approach 1 when $k \to \infty$. Then there exists $\epsilon > 0$ such that $d^k < 1 -$

ϵ for infinitely many k’s.
Thus, the following equation should hold for infinitely many k’s:

$$v^k(s^k) \geq v^k(s^{k-1}, 1) = \frac{k\alpha(r_2 - r_1)}{2} + \sigma^{k-1} \qquad \text{(S-5.1)}$$

By (M.1.7) we get

$$v^k\left(s^{k-1}, d^k\right) - \left[\frac{k\alpha(r_2 - r_1)}{2} + \sigma^{k-1}\right]$$

$$= \left[k\alpha(r_2 - r_1)\int_{d^k}^{1} p \cdot f(p)dp + 2F\left(d^k\right)\left(1 - F\left(d^k\right)\right)\sigma^{k-1} + \left(1 - F\left(d^k\right)\right)^2\sigma^{k-2}\right]$$

$$\cdot \frac{1}{\left(1 - F\left(d^k\right)\right)^2} - \left[\frac{k\alpha(r_2 - r_1)}{2} + \sigma^{k-1}\right]$$

$$= 2\left[k\alpha(r_2 - r_1)\int_{d^k}^{1} p \cdot f(p)dp + 2F\left(d^k\right)\left(1 - F\left(d^k\right)\right)\sigma^{k-1} + \left(1 - F\left(d^k\right)\right)^2\sigma^{k-2}\right]$$

$$\cdot \frac{1}{2\left(1 - F\left(d^k\right)\right)^2} - \frac{\left(1 - F\left(d^k\right)\right)^2\left[k\alpha(r_2 - r_1) + 2\sigma^{k-1}\right]}{2\left(1 - F\left(d^k\right)\right)^2}$$

$$= \left(k\alpha(r_2 - r_1)\left(2\int_{d^k}^{1} p \cdot f(p)dp - \left(1 - F\left(d^k\right)\right)^2\right) - 2\left[1 - F\left(d^k\right)\right]^2\left[\sigma^{k-1} - \sigma^{k-2}\right]\right)$$

$$\cdot \frac{1}{2\left(1 - F\left(d^k\right)\right)^2}$$

The expression $\frac{1}{2\left(1 - F\left(d^k\right)\right)^2}$ is always positive (since $d^k < 1 - \epsilon$), and therefore for infinitely many k’s the following equation holds:

$$k\alpha(r_2 - r_1)\left(2\int_{d^k}^{1} p \cdot f(p)dp - \left(1 - F\left(d^k\right)\right)^2\right) - 2\left[1 - F\left(d^k\right)\right]^2\left[\sigma^{k-1} - \sigma^{k-2}\right] \geq 0$$

We know that:

$$\sigma^{k-1} \geq v^k(s^{k-2}, 1) = \frac{(k-1)\alpha(r_2 - r_1)}{2} + \sigma^{k-2}$$

Thus,

$$\sigma^{k-1} - \sigma^{k-2} \geq \frac{(k-1)\alpha(r_2 - r_1)}{2}$$

And therefore:

$$k\alpha(r_2 - r_1)\left(2\int_{d^k}^{1} p \cdot f(p)dp - \left(1 - F\left(d^k\right)\right)^2\right) - 2\left[1 - F\left(d^k\right)\right]^2\left[\sigma^{k-1} - \sigma^{k-2}\right] \leq$$

$$k\alpha(r_2 - r_1)\left(2\int_{d^k}^{1} p \cdot f(p)dp - \left(1 - F\left(d^k\right)\right)^2\right) - 2\left[1 - F\left(d^k\right)\right]^2\frac{(k-1)\alpha(r_2 - r_1)}{2} =$$

$$\alpha(r_2 - r_1)\left[k\left(2\int_{d^k}^{1} p\cdot f(p)dp - \left(1 - F\left(d^k\right)\right)^2 - \left[1 - F\left(d^k\right)\right]^2\right) + \left[1 - F\left(d^k\right)\right]^2\right] \tag{S-5.2}$$

Let us focus on the first addend:

$$\begin{aligned}
& k\left(2\int_{d^k}^{1} p\cdot f(p)dp - \left(1 - F\left(d^k\right)\right)^2 - \left[1 - F\left(d^k\right)\right]^2\right) \\
= \; & k\left(2\int_{d^k}^{1} p\cdot f(p)dp - \left(1 - F\left(d^k\right)\right)^2 + 1 - 2F\left(d^k\right) + \left(F\left(d^k\right)\right)^2\right) \\
= \; & 2k\left(\int_{d^k}^{1} p\cdot f(p)dp - \left[1 - F\left(d^k\right)\right]\right) \\
= \; & 2k\left(\int_{d^k}^{1} p\cdot f(p)dp - \int_{d^k}^{1} f(p)dp\right)
\end{aligned}$$

Returning to (S-5.2), a necessary condition for (S-5.1) to hold is

$$\alpha(r_2 - r_1)\cdot\left[2k\left(\int_{d^k}^{1} p\cdot f(p)dp - \int_{d^k}^{1} f(p)dp\right) + \left(\int_{d^k}^{1} f(p)dp\right)^2\right] \geq 0$$

and therefore

$$2k \leq \frac{\left(\int_{d^k}^{1} f(p)dp\right)^2}{\int_{d^k}^{1} f(p)dp - \int_{d^k}^{1} p\cdot f(p)dp} \tag{S-5.3}$$

must hold for infinitely many k's. But $d^k \in [0,1-\epsilon]$ and the right side of equation (S-5.3) is a continuous function on the closed interval $[0,1-\epsilon]$ (the denominator can not equal 0, because $0 \leq p < 1$ and because of the definition of $f(p)$). Thus, it has a maximum, and, therefore, the inequality cannot hold for infinitely many k's.

Supplementary Note 6 Validity of the one-stage deviation principle

The one-stage deviation principle states that a strategy-profile s is subgame perfect if and only if there is no player i and no strategy s_i that agrees with s_i except at a single (stage) t and (history) h^t, and such that s_i is a better response to s_{-i} than s_i conditional on history h^t being reached.

Subgame perfection obviously implies the one-stage deviation principle. For the formal proof that the one-stage deviation principle is a sufficient condition for subgame perfection see Fudenberg and Tirole [13] (p. 109). The proof of this principle for finite multi-stage games argues that if a strategy satisfies the one-stage deviation principle then it cannot be improved at some subgame by a finite number of deviations.

This proof is not sufficient in the general case of infinite-horizon games, since it does not exclude the possibility that player i could gain by some infinite sequence of deviations, even though he cannot gain by a single deviation in any subgame. Therefore, generally, the principle holds for an infinite-horizon stage game that is continuous at infinity, i.e. a game in which events in the distant future are relatively unimportant. This condition is satisfied if the overall payoff function is a discounted sum of per-period payoffs, and the per-period payoffs are

uniformly bounded.

However, in our case, although in each stage (E-state) there is a positive probability that the game will continue (and in this sense the game has an infinite horizon), the proof of the finite game applies. Since we have limited ourselves to Markovian strategies that depend only on the E-state number k (i.e. the current resistance level) and not on the history of observed actions, the strategies themselves are finite vectors. Therefore, any alternative strategy s_i contains only a finite number of deviations from s_i, and the One-Stage Deviation Principle can be applied without the use of discounted payoffs.

Supplementary Note 7 Equilibrium Conditions

Based on the One-Stage Deviation Principle, the basic condition for an MPE is stated in (M.3.1). Using equations (M.1.5) and (M.1.7) and basic algebra, the condition (M.3.1) can be also represented as a set of differences-equations.

$v_i^k\left(s^{k-1}, d^k, d^k\right) \geq v_i^k \;\; s^{k-1}, d^k, d^k$ if for every $k = 1, \dots, M$ and for all d^k

$$\left(1-(F(d^k))^2\right) \cdot k\alpha(r_2-r_1)\int_{d^k}^{1} p \cdot f(p)dp - \;\; 1-F(d^k)F(d^k) \;\; \cdot k\alpha(r_2-r_1)\int_{d^k}^{1} p \cdot f(p)dp$$
$$\leq \left(1-F(d^k)\right)^2 \;\; F(d^k)-F(d^k) \;\;\; v_i^{k-1}\left(s^{k-1}\right) - v_i^{k-2}\left(s^{k-2}\right) \tag{S-7.1}$$

Thus, when $k = 1$:

$$1-\left[F\left(d^k\right)\right]^2 \;\; \int_{d^k}^{1} p \cdot f(p)dp - \left(1-F\left(d^k\right)F \;\; d^k \;\;\right)\int_{d^k}^{1} p \cdot f(p)dp \leq 0 \tag{S-7.2}$$

For any given $0 \leq d^k < 1$ let

$$g_{d^k} \;\; d^k \;\; = \frac{1-\left[F\left(d^k\right)\right]^2 \;\; {}_{d^k}^{1} p \cdot f(p)dp - \left(1-F\left(d^k\right)F \;\; d^k \;\;\right) \; {}_{d^k}^{1} p \cdot f(p)dp}{1-F\left(d^k\right) \;\; ^2\left(F\left(d^k\right)-F \;\; d^k \;\;\right)}$$

Using this definition, the differences-equations for $k \geq 2$ become:

when $k = 2$:

for $d^k < d^k$, $v_i^k\left(s^{k-1}, d^k, d^k\right) \geq v_i^k \;\; s^{k-1}, d^k, d^k$ if

$$g_{d^k} \;\; d^k \;\; \leq \frac{v_i^{k-1}(s^{k-1})}{k\alpha(r_2-r_1)} \tag{S-7.3}$$

and for $d^k > d^k$, $v_i^k\left(s^{k-1}, d^k, d^k\right) \geq v_i^k \;\; s^{k-1}, d^k, d^k$ if

$$g_{d^k}\;\; d^k \;\; \geq \frac{v_i^{k-1}(s^{k-1})}{k\alpha(r_2 - r_1)} \tag{S-7.4}$$

and when $k \geq 3$:
for $d^k < d^k$, $v_i^k\left(s^{k-1}, d^k, d^k\right) \geq v_i^k \;\; s^{k-1}, d^k, d^k$ if

$$g_{d^k}\;\; d^k \;\; \leq \frac{v_i^{k-1}(s^{k-1}) - v_i^{k-2}(s^{k-2})}{k\alpha(r_2 - r_1)} \tag{S-7.5}$$

and for $d^k > d^k$, $v_i^k\left(s^{k-1}, d^k, d^k\right) \geq v_i^k \;\; s^{k-1}, d^k, d^k$ if

$$g_{d^k}\;\; d^k \;\; \geq \frac{v_i^{k-1}(s^{k-1}) - v_i^{k-2}(s^{k-2})}{k\alpha(r_2 - r_1)} \tag{S-7.6}$$

Supplementary Note 8 Lemma M.3.1

Lemma M.3.1: $g_{d^k}\;\; d^k$ is strictly increasing:

$$g_{d^k}\;\; d^k \;\; > g_{d^k}\;\; d^k - \epsilon \;\;, \qquad \forall 0 \leq d^k < 1, \qquad 0 < \epsilon < d$$

Proof.

$g_{d^k}\;\; d^k$ is not well defined at $d^k = d^k$. Therefore, we first show monotonicity for $d^k > d^k$ and $d^k < d^k$, and then we show that the limit $\lim_{d^k \to d^k} g_{d^k}\;\; d^k$ exists.

We need to show that for any $d^k < x - \epsilon < x$ or $x - \epsilon < x < d^k$:

$$g_{d^k}(x) - g_{d^k}(x - \epsilon) > 0$$

$g_{d^k}(x) - g_{d^k}(x - \epsilon) =$

$$\frac{1-\left[F\left(d^k\right)\right]^2 \;\; {}_x^1 p\cdot f(p)dp - \;\; 1-F\left(d^k\right)F(x) \;\; {}_{d^k}^1 p\cdot f(p)dp}{1-F\left(d^k\right) \;\; ^2 \;\; F\left(d^k\right)-F(x)}$$

$$-\frac{1-\left[F\left(d^k\right)\right]^2 \;\; {}_{x-\epsilon}^1 p\cdot f(p)dp - \;\; 1-F\left(d^k\right)F(x-\epsilon) \;\; {}_{d^k}^1 p\cdot f(p)dp}{1-F\left(d^k\right) \;\; ^2 \;\; F\left(d^k\right)-F(x-\epsilon)}$$

The common denominator is:

$$1 - F\left(d^k\right) \quad ^{2} \quad F\left(d^k\right) - F(x) \quad F\left(d^k\right) - F(x-\epsilon) \quad > 0$$

It is positive when either $d^k < x - \epsilon < x$ or $x - \epsilon < x < d^k$. Thus, in these cases we need to show that the nominator is positive as well.

$$\left[F\left(d^k\right) - F(x-\epsilon)\right] \quad \cdot \quad 1 - \left[F\left(d^k\right)\right]^2 \int_x^1 p \cdot f(p)dp - \quad 1 - F\left(d^k\right)F(x) \quad \int_{d^k}^1 p \cdot f(p)dp\Bigg]$$

$$-\left[F\left(d^k\right) - F(x)\right] \cdot \quad 1 - \left[F\left(d^k\right)\right]^2 \int_{x-\epsilon}^1 p \cdot f(p)dp - \quad 1 - F\left(d^k\right)F(x-\epsilon) \quad \int_{d^k}^1 p \cdot f(p)dp\Bigg]$$

$$= \quad 1 - \left[F\left(d^k\right)\right]^2 \quad \left(\left[F\left(d^k\right) - F(x-\epsilon)\right]\int_x^1 p \cdot f(p)dp - \left[F\left(d^k\right) - F(x)\right]\int_{x-\epsilon}^1 p \cdot f(p)dp\right)$$

$$- \quad {}_{d^k}^{1}\, p \cdot f(p)dp\left[1 - F\left(d^k\right)F(x)\right]\left(\left[F\left(d^k\right) - F(x-\epsilon)\right] - \left[1 - F\left(d^k\right)F(x-\epsilon)\right]\left[F\left(d^k\right) - F(x)\right]\right)$$

$$= \quad 1 - \left[F\left(d^k\right)\right]^2 \quad \left(\left[F\left(d^k\right) - F(x-\epsilon)\right]\int_x^1 p \cdot f(p)dp - \left[F\left(d^k\right) - F(x)\right]\int_{x-\epsilon}^1 p \cdot f(p)dp\right)$$

$$- \quad {}_{d^k}^{1}\, p \cdot f(p)dp \quad 1 - \left[F\left(d^k\right)\right]^2 \quad \left[F(x) - F(x-\epsilon)\right]$$

$$= \quad 1 - \left[F\left(d^k\right)\right]^2 \quad \Bigg(\left[F\left(d^k\right) - F(x-\epsilon)\right]\int_x^1 p \cdot f(p)dp - \left[F\left(d^k\right) - F(x)\right]\int_{x-\epsilon}^1 p \cdot f(p)dp$$

$$- \left[F(x) - F(x-\epsilon)\right]\int_{d^k}^1 p \cdot f(p)dp\Bigg)$$

At this stage we need to examine each case separately.

When $x - \epsilon < x < d^k$:

$$= \quad 1 - \left[F\left(d^k\right)\right]^2 \quad \Bigg(\int_{d^k}^1 p \cdot f(p)dp\left(\left[F\left(d^k\right) - F(x-\epsilon)\right] - \left[F\left(d^k\right) - F(x)\right] - \left[F(x) - F(x-\epsilon)\right]\right)$$

$$+ \int_x^{d^k} p \cdot f(p)dp\left(\left[F\left(d^k\right) - F(x-\epsilon)\right] - \left[F\left(d^k\right) - F(x)\right]\right) - \int_{x-\epsilon}^{x} p \cdot f(p)dp\left[F\left(d^k\right) - F(x)\right]$$

$$= \quad 1 - \left[F\left(d^k\right)\right]^2 \quad \Bigg(\int_x^{d^k} p \cdot f(p)dp\left[F(x) - F(x-\epsilon)\right] - \int_{x-\epsilon}^{x} p \cdot f(p)dp\left[F\left(d^k\right) - F(x)\right]$$

The first multiplicand is always positive (since $d^k < 1$), and the second multiplicand equals:

$$\int_{x-\epsilon}^{x} f(t)dt \cdot \int_x^{d^k} p \cdot f(p)dp - \int_{x-\epsilon}^{x} t \cdot f(t)dt \cdot \int_x^{d^k} f(p)dp$$

$$= \int_{x-\epsilon}^{x} \quad \int_x^{d^k} f(t)f(p)pdp\Bigg] dt - \int_{x-\epsilon}^{x} \quad \int_x^{d^k} f(t)f(p)tdp\Bigg] dt > 0$$

and when $d^k < x - \epsilon < x$:

$$= \ 1 - \left[F\left(d^k\right)\right]^2 \ \ \left(\int_x^1 p \cdot f(p)dp \left(\left[F\left(d^k\right) - F(x-\epsilon)\right] - \left[F\left(d^k\right) - F(x)\right] - [F(x) - F(x-\epsilon)]\right)\right.$$

$$+ \int_{x-\epsilon}^{x} p \cdot f(p)dp \left([F(x-\epsilon) - F(x-)] + \left[F(x) - F\left(d^k\right)\right]\right)$$

$$\left. - \int_{d^k}^{x-\epsilon} p \cdot f(p)dp \, [F(x) - F(x-\epsilon)]\right)$$

$$= \ 1 - \left[F\left(d^k\right)\right]^2 \ \ \left(\int_{x-\epsilon}^{x} p \cdot f(p)dp \left[F(x-\epsilon) - F\left(d^k\right)\right] - \int_{d^k}^{x-\epsilon} p \cdot f(p)dp \, [F(x) - F(x-\epsilon)]\right)$$

The first multiplicand is always positive (since $d^k < 1$), and the second multiplicand equals:

$$\int_{d^k}^{x-\epsilon} f(t)dt \cdot \int_{x-\epsilon}^{x} p \cdot f(p)dp - \int_{d^k}^{x-\epsilon} t \cdot f(t)dt \cdot \int_{x-\epsilon}^{x} f(p)dp$$

$$= \int_{d^k}^{x-\epsilon} \quad \int_{x-\epsilon}^{x} f(t)f(p)pdp \Big] \, dt - \int_{d^k}^{x-\epsilon} \quad \int_{x-\epsilon}^{x} f(t)f(p)tdp \Big] \, dt > 0$$

As for $d^k = d^k$:

$$\lim_{d^k \to d^k} g_{d^k} \ \ d^k$$

$$= \lim_{d^k \to d^k} \frac{1 - \left[F\left(d^k\right)\right]^2 \quad {}_{d^k}^{1} p \cdot f(p)dp - \left(1 - F\left(d^k\right) F \ \ d^k \quad\right) \ {}_{d^k}^{1} \ p \cdot f(p)dp}{\left[1 - F\left(d^k\right)\right]^2 \ \ F\left(d^k\right) - F \ \ d^k}$$

$$= \lim_{d^k \to d^k} \frac{1 - \left[F\left(d^k\right)\right]^2 \quad {}_{d^k}^{d^k} \ p \cdot f(p)dp}{\left[1 - F\left(d^k\right)\right]^2 \ \ F\left(d^k\right) - F \ \ d^k}$$

$$- \frac{\left(1 - F\left(d^k\right) F \ \ d^k \quad\right) - \ \ 1 - \left[F\left(d^k\right)\right]^2 \ \Big] \ \ {}_{d^k}^{1} \ p \cdot f(p)dp}{\left[1 - F\left(d^k\right)\right]^2 \ \ F\left(d^k\right) - F \ \ d^k}$$

$$= \lim_{d^k \to d^k} \frac{\left[1 - F\left(d^k\right)\right]\left[1 + F\left(d^k\right)\right] \quad {}_{d^k}^{d^k} \ p \cdot f(p)dp}{\left[1 - F\left(d^k\right)\right]^2 \ \ F\left(d^k\right) - F \ \ d^k} - \frac{F\left(d^k\right) \left(F\left(d^k\right) - F \ \ d^k \quad\right) \ \ {}_{d^k}^{1} \ p \cdot f(p)dp}{\left[1 - F\left(d^k\right)\right]^2 \ \ F\left(d^k\right) - F \ \ d^k}$$

$$= \lim_{d^k \to d^k} \frac{\left[1 + F\left(d^k\right)\right] \quad {}_{d^k}^{d^k} \ p \cdot f(p)dp}{\left[1 - F\left(d^k\right)\right] \quad {}_{d^k}^{d^k} \ f(p)dp} - \frac{F\left(d^k\right) \ \ {}_{d^k}^{1} \ p \cdot f(p)dp}{\left[1 - F\left(d^k\right)\right]^2}$$

$$= \frac{1 + F\left(d^k\right)}{1 - F\left(d^k\right)} d^k - \frac{F\left(d^k\right) \ \ {}_{d^k}^{1} \ p \cdot f(p)dp}{\left[1 - F\left(d^k\right)\right]^2}$$

and therefore the limit value exists.

Supplementary Note 9 Theorem M.3.2

Theorem M.3.2: It is impossible that the following two conditions hold simultaneously at any given E-state $k > 1$*:*

$$v_i^k\left(s^{k-1}, d^k, d^k\right) \leq v_i^k\left(s^{k-1}, d^k - x, d^k\right) for \quad some \quad 0 < x \leq d^k$$

and

$$v_i^k\left(s^{k-1}, d^k, d^k\right) \leq v_i^k\left(s^{k-1}, d^k + y, d^k\right) for \quad some \quad 0 < y < 1 - d^k$$

Proof. When $k > 2$, by (S-7.5), if $v_i^k(s^{k-1}, d^k, d^k) \leq v_i^k(s^{k-1}, d^k - x, d^k)$ then

$$g_{d^k}\left(d^k - x\right) \geq \frac{v_i^{k-1}(s^{k-1}) - v_i^{k-2}(s^{k-2})}{k\alpha(r_2 - r_1)}$$

And by (S-7.6), if $v_i^k(s^{k-1}, d^k, d^k) \leq v_i^k(s^{k-1}, d^k + y, d^k)$ then

$$g_{d^k}\left(d^k + y\right) \leq \frac{v_i^{k-1}(s^{k-1}) - v_i^{k-2}(s^{k-2})}{k\alpha(r_2 - r_1)}$$

Combining these equations, we get

$$g_{d^k}\left(d^k - x\right) \geq g_{d^k}\left(d^k + y\right)$$

But by lemma 5.1 g is strictly increasing.
The proof for $k = 2$ follows the same steps, using (S-7.3) and (S-7.4) instead of (S-7.5) and (S-7.6).

Supplementary Note 10 Lemma M.3.3

Lemma M.3.3: $\exists 0 < \epsilon,$ $\lim_{d^k \to 1} v_i^k\left(s^{k-1}, d^k - \epsilon, d^k\right) > v_i^k\left(s^{k-1}, d^k, d^k\right)$

Proof. By lemma M.2.1

$$v_i^k\left(s^{k-1}, d^k, d^k\right) = \frac{k\alpha(r_2 - r_1)}{2} + v_i^{k-1}\left(s^{k-1}\right)$$

By (M.1.5)

$$
\begin{aligned}
&\lim_{d^k \to 1} v_i^k(s^{k-1}, d^k - \epsilon, d^k) \\
&\quad = \Bigg(k\alpha(r_2 - r_1) \int_{d^k-\epsilon}^{1} p \cdot f(p)dp \\
&\quad + \ F\left(d^k - \epsilon\right) \ 1 - F\left(d^k\right) \ + \ 1 - F\left(d^k - \epsilon\right) \ F\left(d^k\right) \ v_i^{k-1}(s^{k-1}) \\
&\quad + \ 1 - F\left(d^k - \epsilon\right) \quad 1 - F\left(d^k\right) \ v_i^{k-2}(s^{k-2}) \Bigg) \cdot \frac{1}{1 - F\left(d^k - \epsilon\right) F\left(d^k\right)} \\
&\quad = \frac{k\alpha(r_2 - r_1) \ \int_{d^k-\epsilon}^{1} p \cdot f(p)dp + \ 1 - F\left(d^k - \epsilon\right) \ v_i^{k-1}(s^{k-1})}{1 - F\left(d^k - \epsilon\right)}
\end{aligned}
$$

Thus, we need to show that

$$
\frac{k\alpha(r_2 - r_1)}{2} + v_i^{k-1}(s^{k-1}) - \frac{k\alpha(r_2 - r_1) \ \int_{d^k-\epsilon}^{1} p \cdot f(p)dp + \ 1 - F\left(d^k - \epsilon\right) \ v_i^{k-1}(s^{k-1})}{1 - F\left(d^k - \epsilon\right)} < 0
$$

Checking this condition:

$$
\begin{aligned}
&\tfrac{k\alpha(r_2-r_1)}{2} + v_i^{k-1}(s^{k-1}) - \tfrac{k\alpha(r_2-r_1) \ \int_{d^k-\epsilon}^{1} p\cdot f(p)dp + \ 1-F\left(d^k-\epsilon\right) \ v_i^{k-1}(s^{k-1})}{1-F\left(d^k-\epsilon\right)} \\
&\quad = \tfrac{k\alpha(r_2-r_1)}{2} - \tfrac{k\alpha(r_2-r_1) \ \int_{d^k-\epsilon}^{1} p\cdot f(p)dp}{1-F\left(d^k-\epsilon\right)} \\
&\quad = k\alpha(r_2 - r_1) \ \tfrac{1}{2} - \tfrac{\int_{d^k-\epsilon}^{1} p\cdot f(p)dp}{\int_{d^k-\epsilon}^{1} f(p)dp} \Bigg] \\
&\quad > k\alpha(r_2 - r_1) \ \tfrac{1}{2} - \tfrac{\int_{d^k-\epsilon}^{1} \left(d^k-\epsilon\right)\cdot f(p)dp}{\int_{d^k-\epsilon}^{1} f(p)dp} \Bigg] \\
&\quad = k\alpha(r_2 - r_1)\left[\tfrac{1}{2} - \left(d^k - \epsilon\right)\right]
\end{aligned}
$$

And $k\alpha(r_2 - r_1)\left[\frac{1}{2} - \left(d^k - \epsilon\right)\right] < 0$ for any ϵ such that $0 < \epsilon < \left(d^k - 0.5\right)$.

Supplementary Note 11 Theorem M.3.5

Theorem M.3.5: Let (d^k, d^k) be a stage equilibrium.

$$
\lim_{k \to \infty} d^k \neq 1
$$

Proof. When $k \geq 3$, by *(S-7.5)* a player does not have an incentive to deviate downwards from (d^k, d^k) if for any $d^k < d^k$,

$$
g_{d^k} \ d^k \ \leq \frac{v_i^{k-1}(s^{k-1}) - v_i^{k-2}(s^{k-2})}{k\alpha(r_2 - r_1)}
$$

and by (S-7.6), a player does not have an incentive to deviate upwards from (d^k, d^k) if for any $d^k > d^k$,

$$g_{d^k}\ \ d^k\ \ \geq \frac{v_i^{k-1}(s^{k-1})-v_i^{k-2}(s^{k-2})}{k\alpha(r_2-r_1)}$$

Therefore, due to the monotonicity of g (lemma 5.1), (d^k, d^k) is a stage equilibrium if

$$g_{d^k}\left(d^k\right) = \frac{v_i^{k-1}(s^{k-1})-v_i^{k-2}(s^{k-2})}{k\alpha(r_2-r_1)}$$

We start with the term on the right-hand side of the equation. The maximal possible immediate payoff (with perfect information) at E-state k is $k\alpha(r_2 - r_1)$, and therefore

$$\lim_{k\to\infty} \frac{v_i^{k-1}(s^{k-1})-v_i^{k-2}(s^{k-2})}{k\alpha(r_2-r_1)} \leq \lim_{k\to\infty} \frac{(k-1)\alpha(r_2-r_1)}{k\alpha(r_2-r_1)} = 1$$

As to the left term, by (M.3.2) for any k,

$$g_{d^k}(d^k) = \frac{1+F\left(d^k\right)}{1-F\left(d^k\right)} d^k - \frac{F\left(d^k\right)\ \ {}_{d^k}^{1} p\cdot f(p)dp}{\left[1-F\left(d^k\right)\right]^2} = \frac{1+F\ \ \tilde{d}^k}{1-F\ \ \tilde{d}^k}\tilde{d}^k - \frac{F\ \ \tilde{d}^k}{1-F\left(d^k\right)} \cdot \frac{{}_{\tilde{d}^k}^{1} p\cdot f(p)dp}{{}_{\tilde{d}^k}^{1} f(p)dp}$$

and therefore

$$\lim_{d^k\to 1} g_{d^k}(d^k) = \lim_{d^k\to 1} \frac{1+F\left(d^k\right)}{1-F\left(d^k\right)} d^k - \frac{F\left(d^k\right)}{1-F\left(d^k\right)} \cdot \frac{{}_{d^k}^{1} p\cdot f(p)dp}{{}_{d^k}^{1} f(p)dp}$$

The limit of the last part of this term is:

$$\lim_{d^k\to 1} \frac{{}_{d^k}^{1} p\cdot f(p)dp}{{}_{d^k}^{1} f(p)dp} = 1$$

and therefore

$$\lim_{d^k\to 1} g_{d^k} = \lim_{d^k\to 1} \frac{2+1-1}{1-F\left(d^k\right)} = \lim_{d^k\to 1} \frac{1}{1-F\left(d^k\right)} = \infty$$

which proves that it cannot equal $\lim_{k\to\infty} \frac{v_i^{k-1}(s^{k-1})-v_i^{k-2}(s^{k-2})}{k\alpha(r_2-r_1)} \leq 1$ and therefore it can not be a symmetric stage equilibrium decision rule.

Supplementary Note 12 Additional Examples of Theoretical Information Distributions

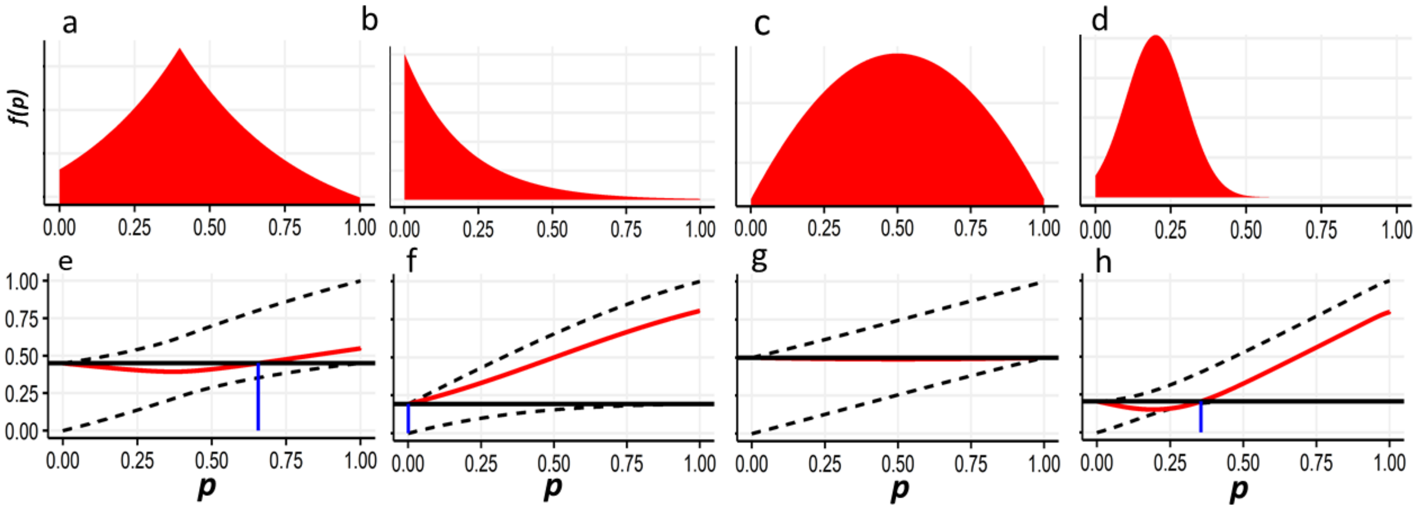

Supplementary Figure 1: Theoretical posterior distributions of bacterial infection signals, and corresponding threshold values possible by coarsening the data, as in Figure 2 in the main text. (a-d) Probability distribution functions $f(p)$ representing distinct scenarios of a bacterial infection signal. (e-h) Corresponding (top-bottom) values of E (expected posterior value, horizontal black lines), P_H and P_L (posterior probability of bacterial infection given a high and low signal, upper and lower dashed curves, respectively), their difference $P_H - P_L$ (red curves) and the minimal threshold T (blue vertical lines) achievable by coarsening the data. Distributions are as follows: truncated double exponential with scale 0.3 and scale 0.3 (a,e); truncated exponential with rate 5 (b,f); truncated normal with mean 0.5 and standard deviation 1 (c,g); and truncated normal with mean 0.2 and standard deviation 0.05.
