# Supplementary material for: A game theoretic approach reveals that discretizing clinical information can reduce antibiotic misuse
Source: Nat Commun. 2021 Feb 19;12:1148. doi: 10.1038/s41467-021-21088-5 (PMC7895914; doi:10.1038/s41467-021-21088-5)
Supplement: Supplementary file 2 — Reporting Summary [file 41467_2021_21088_MOESM2_ESM.pdf]

nature research

Corresponding author(s): Uri Obolski

Last updated by author(s): Jan 3, 2021

Reporting Summary

Nature Research wishes to improve the reproducibility of the work that we publish. This form provides structure for consistency and transparency in reporting. For further information on Nature Research policies, see our Editorial Policies and the Editorial Policy Checklist.

Statistics

For all statistical analyses, confirm that the following items are present in the figure legend, table legend, main text, or Methods section.

n/a	Confirmed	
☐	☒	The exact sample size (*n*) for each experimental group/condition, given as a discrete number and unit of measurement
☒	☐	A statement on whether measurements were taken from distinct samples or whether the same sample was measured repeatedly
☒	☐	The statistical test(s) used AND whether they are one- or two-sided<br>*Only common tests should be described solely by name; describe more complex techniques in the Methods section.*
☒	☐	A description of all covariates tested
☒	☐	A description of any assumptions or corrections, such as tests of normality and adjustment for multiple comparisons
☒	☐	A full description of the statistical parameters including central tendency (e.g. means) or other basic estimates (e.g. regression coefficient) AND variation (e.g. standard deviation) or associated estimates of uncertainty (e.g. confidence intervals)
☒	☐	For null hypothesis testing, the test statistic (e.g. *F*, *t*, *r*) with confidence intervals, effect sizes, degrees of freedom and *P* value noted<br>*Give P values as exact values whenever suitable.*
☒	☐	For Bayesian analysis, information on the choice of priors and Markov chain Monte Carlo settings
☒	☐	For hierarchical and complex designs, identification of the appropriate level for tests and full reporting of outcomes
☒	☐	Estimates of effect sizes (e.g. Cohen's *d*, Pearson's *r*), indicating how they were calculated

Our web collection on statistics for biologists contains articles on many of the points above.

Software and code

Policy information about availability of computer code

Data collection	Data were collected using Microsoft Excel (2016), Microsoft Corporation
Data analysis	Data were analyzed using R 3.6.1, R Core Team (2020)

For manuscripts utilizing custom algorithms or software that are central to the research but not yet described in published literature, software must be made available to editors and reviewers. We strongly encourage code deposition in a community repository (e.g. GitHub). See the Nature Research guidelines for submitting code & software for further information.

Data

Policy information about availability of data

All manuscripts must include a data availability statement. This statement should provide the following information, where applicable:

- Accession codes, unique identifiers, or web links for publicly available datasets
- A list of figures that have associated raw data
- A description of any restrictions on data availability

Raw data were generated at Hillel Yaffe Medical Center. Derived data supporting the findings of this study are available upon reasonable request from the corresponding author.

Field-specific reporting

Life sciences study design

All studies must disclose on these points even when the disclosure is negative.

Sample size	Sample size was determined based on all patient files available to us. Since we perform no inference on the data but rather use descriptive analysis as an example of a distribution, no sample size calculation was performed.
Data exclusions	No data were excluded beyond the selection criteria described in the paper.
Replication	Since we perform no inference on the data but rather use descriptive analysis as an example of a distribution for a specific setting, this is not relevant.
Randomization	Not relevant to our study - the data were taken retrospectively, using all files available to us.
Blinding	Not relevant to our study - the data were taken retrospectively, using all files available to us and analysis was descriptive, so no blinding was necessary.

Reporting for specific materials, systems and methods

We require information from authors about some types of materials, experimental systems and methods used in many studies. Here, indicate whether each material, system or method listed is relevant to your study. If you are not sure if a list item applies to your research, read the appropriate section before selecting a response.

Materials & experimental systems

n/a	Involved in the study	
☒	☐	Antibodies
☒	☐	Eukaryotic cell lines
☒	☐	Palaeontology and archaeology
☒	☐	Animals and other organisms
☒	☐	Human research participants
☐	☒	Clinical data
☒	☐	Dual use research of concern

Methods

n/a	Involved in the study	
☒	☐	ChIP-seq
☒	☐	Flow cytometry
☒	☐	MRI-based neuroimaging

Clinical data

Policy information about clinical studies

All manuscripts should comply with the ICMJEguidelines for publication of clinical research and a completedCONSORT checklist must be included with all submissions.

Clinical trial registration	NA - not a clinical trial, retrospective data
Study protocol	NA -not a clinical trial, retrospective data
Data collection	We obtained EMRs of 1202 children aged < 2 years were hospitalized for bronchiolitis at Hillel Yaffe Medical Center in Hadera, Israel between 2008-2018. Selection of EMRs was based on all children who tested positive for RSV by antigen detection enzyme immunoassay in the time period.
Outcomes	All children tested positive for RSV by antigen detection enzyme immunoassay, but 967 had only RSV bronchiolitis (viral infection), whereas 235 also had bacterial pneumonia, as confirmed in an in an X-ray scan. The only outcome used in the paper was viral infection Vs. viral+bacterial infection.
